# Fast or Frugal, but Not Both: Decision Heuristics Under Time Pressure

**DOI:** 10.1037/xlm0000419

**Published:** 2017-05-29

**Authors:** Sebastian Bobadilla-Suarez, Bradley C. Love

**Affiliations:** 1 Department of Experimental Psychology, University College London, and The Alan Turing Institute, London, United Kingdom

**Keywords:** heuristics, reaction time (RT), selective attention, search costs, decision making

## Abstract

Heuristics are simple, yet effective, strategies that people use to make decisions. Because heuristics do not require all available information, they are thought to be easy to implement and to not tax limited cognitive resources, which has led heuristics to be characterized as fast-and-frugal. We question this monolithic conception of heuristics by contrasting the cognitive demands of two popular heuristics, Tallying and Take-the-Best. We contend that heuristics that are frugal in terms of information usage may not always be fast because of the attentional control required to implement this focus in certain contexts. In support of this hypothesis, we find that Take-the-Best, while being more frugal in terms of information usage, is slower to implement and fares worse under time pressure manipulations than Tallying. This effect is then reversed when search costs for Take-the-Best are reduced by changing the format of the stimuli. These findings suggest that heuristics are heterogeneous and should be unpacked according to their cognitive demands to determine the circumstances a heuristic best applies.

Some decision procedures are slow and information demanding whereas others are “fast and frugal” (Gigerenzer & Gaissmaier, 2011), though for a dissenting opinion on the prevalence of heuristic use see Newell, Weston, and Shanks (2003), and Newell (2005). Consider a scenario in which a child suddenly crosses the street to get his ball. The driver has less than a second to evaluate the situation and decide whether to press hard on the brakes without swerving or press on the brakes and swerve onto the sidewalk. The former option risks hitting the child and the latter option risks hitting other pedestrians. The optimal decision depends on cues such as the speed of the vehicle, the distance from the car to other people, the car’s stopping distance, the number of people on the sidewalk, the driver’s ability, and so forth. What is the best way to integrate all this information quickly? Representing and integrating all this information in an optimal manner may be impossible or too time-consuming.

Alternatives to the computationally intensive decision strategies are commonly referred to as fast and frugal heuristics (Todd & Gigerenzer, 2000). Heuristics are fast in that they can be applied quickly and frugal in the sense that they use less information to make a decision than more complex procedures that selectively weigh all information sources. Despite the fact that heuristics use less information from the environment, in practice they can perform very well, often surpassing regression approaches for certain decision problems (Czerlinski, Gigerenzer, & Goldstein, 1999). Heuristics have been described as “efficient cognitive processes, conscious or unconscious, that ignore part of the information” (Gigerenzer & Gaissmaier, 2011) and as using “a minimum of time, knowledge, and computation to make adaptive choices in real environments” (Todd & Gigerenzer, 2000). This characterization of heuristics differs from earlier accounts that cast heuristics as imperfect approximations of rational decision procedures (Tversky & Kahneman, 1974).

When complex decision strategies, such as multiple linear regression, cannot be implemented because of resource constraints, such as lack of time, people might use alternative strategies like heuristics (Todd & Gigerenzer, 2000). In this study, we considered whether two popular heuristics differ from one another in cognitive processing requirements as reflected by timing constraints. Specifically, we compared two popular heuristics, Tallying (TAL) and Take-the-Best (TTB), which we introduce by way of example below. These heuristics follow from previous work, such as the lexicographic heuristics that only take into account the most discriminating attribute value (Fishburn, 1967; Tversky, 1969), the majority of confirming dimensions heuristic (Russo & Dosher, 1983), and the equal weights strategy (Dawes, 1979).

Suppose one wants to predict whether China or India will have higher gross domestic product (GDP) growth based on their productive capabilities, natural resource wealth, and the diversity in their exports, and so forth. The TAL heuristic chooses the country that bests the other across the most measures. TAL does not selectively weigh cues as linear regression does, but instead merely counts the number of cues favoring one alternative over the other (Gigerenzer & Gaissmaier, 2011). On the other hand, the TTB heuristic chooses based on the most predictive cue and only considers the next best cue when there is a tie. TTB implies that cues are rank ordered in terms of their predictive validity in determining the criterion (e.g., in predicting China or India). TTB sequentially searches until a discriminating cue is found and, thus, may reach a decision after only considering the first best cue. These two heuristics can both be effective in practice, but can differ in their choices as shown in an example trial of our first experiment in Figure 1.[Fig-anchor fig1]

We hypothesize that these two heuristics differ in their cognitive processing requirements such that what is fast and what is frugal is contingent on the cognitive processes invoked by the environment. In Experiment 1, we predict that TTB will fare worse under time pressure than TAL, whereas we predict the opposite pattern in Experiment 2. Although one might expect TTB to be fast given that it samples very little information (Bröder & Gaissmaier, 2007; Khader et al., 2011), the cognitive demands in Experiment 1 should be high for TTB users because the stimulus format invites an effortful sequential search procedure. In contrast, the stimulus format in Experiment 2 reduces this search burden while making it more difficult for TAL users to perform rapid summation operations.

Our experimental procedures are intended to expand the scope of inquiry by deviating from the original formulation of TTB (Gigerenzer & Todd, 1999) in which decisions were made from memory for environments; instead our method invites participants to make inference from givens in the environment. Although memory demands are an important aspect of heuristic application, our studies focus more on the attentional demands of applying heuristics. Related previous efforts have noted that one subtle complexity of TTB is that it requires a hierarchy of cue validities (Dougherty, Franco-Watkins, & Thomas, 2008; Juslin & Persson, 2002) and that noncompensatory strategies such as TAL can be affected by cue salience (Platzer & Bröder, 2012).

When compared with algorithms that are more computationally intensive such as linear regression, TAL and TTB have various algorithmic aspects in common. Their most salient similarity is that they both disregard covariance structure among cues (Parpart, Jones & Love, under revision). Both heuristics also disregard relative cue weight magnitudes.

Despite these similarities, these two heuristics may differ in their cognitive demands. For instance, TTB implements a search for the best discriminating cue that has been argued to take time (Bröder & Gaissmaier, 2007) and a certain level of cognitive control because of selective attention to the relevant cue, as well as inhibition of the irrelevant ones. Such sequential control processes are thought to be effortful, serial in nature, and time-consuming (Posner & Presti, 1987). In effect, TTB has the prescription that people will embark on a serial search, which in our first experiment is a visually guided search. Such visually guided searches are a common domain for research on top-down attentional control mechanisms (Mozer & Baldwin, 2008; Wolfe, Cave, & Franzel, 1989).

TALs cognitive requirements may be quite different than those used for TTB. We argue that TAL requires people’s ability to do quick summations over stimuli. Related research on numerosity has shown that people can be very fast at doing these types of operations without explicit counting (Feigenson, Dehaene, & Spelke, 2004; Mandler & Shebo, 1982). Given the right representation format of cues, a TAL decision problem could be reduced to a low-level perceptual categorization problem (e.g., Palmer, Huk, & Shadlen, 2005). However, if the representation format is not suitable for such operations, as we manipulate in Experiment 2, we predict that TALs performance should be reduced.

In Experiment 1, we predicted that compliance with the TAL heuristic would be higher than for TTB under time pressure conditions even though TAL considers more aspects of the stimulus. In Experiment 2, we attempted to reverse this effect by eliminating search costs for TTB and altering the stimulus format in a manner that obstructs quick summation operations that favor TAL performance. Together, these two studies aim to establish that heuristics should be understood not only in terms of how they perform in various information environments, but also in terms of the cognitive processes they engage.

## Experiment 1

In Experiment 1, we test the hypothesis that the TTB heuristic will not always lead to rapid decisions because of the search costs and attentional control it can demand. For certain stimulus formats, it should be possible for TAL to be faster than TTB. For example, color-coded stimulus values should allow for rapid perceptual integration of cue values, making TAL faster than TTB. Furthermore, TAL use should be unaffected by the randomization of cue position across trials because TAL treats all cues identically and summates. In contrast, TTBs search requirements should be increased by this randomization and should not benefit from the color coding. Thus, we predict that TAL should be faster than TTB under these conditions because of the basic resource requirements of each heuristic.

### Method

#### Participants

Participants (206 total, 95 female) were recruited on Amazon Mechanical Turk, an online study platform commonly used in psychological studies with good results (Crump, McDonnell, & Gureckis, 2013). Participants were restricted to the United States of America and assigned either to the TAL condition (107 total, 58 female) or the TTB condition (99 total, 39 female). They received $2.50 to complete a 40 min (approx.). learning and decision making task and the best participant was offered a $20 bonus in each condition. The average age of participants was 37.9 years (*SD* = 12.56). The study was approved by the local UCL ethics committee.

#### Design and materials

As a between participants manipulation, participants were explicitly instructed to use either TAL or TTB in a two-alternative forced choice task (i.e., choosing which country will have higher GDP). Each participant first completed a *practice* phase (72 trials) followed by a *test* phase, consisting of two blocks of trials. Whether the test block was self-paced (72 trials) or speeded (72 trials) was counterbalanced across trials. Trial order was randomized separately for each participant.

Within participants, the same 72 trials were used across these three (practice, self-paced test, and speeded test) experimental segments. Trials were designed such that one response was consistent with TAL and the other with TTB. In other words, the heuristics disagreed on every trial, which allowed for discriminating heuristic use. Perfect performance is achievable for both heuristics because we measured compliance with an instructed heuristic (compliance with heuristic is understood as percent correct). Conversely, expected performance under random responding was at chance (50%). The presentation order of the cues was randomized on a trial-by-trial basis.

The 72 trials for each experimental segment consisted of three trial types in terms of three difficulty levels. The 72 trials were equally partitioned into the three trial types resulting in 24 trials for each difficulty level. Difficulty was defined differently for each heuristic. For TTB, difficulty level is referred to as Q1 (cue 1), Q2 (cue 2), and Q3 (cue 3), in order of ascending difficulty. Q1 trials represent trials where retrieving the value for the best cue was sufficient to respond in compliance with TTB. Similarly, Q2 trials require retrieving the value for the second-best cue and Q3 trials require retrieving the value for the third-best cue. For TAL, difficulty level is referred to as Δ3 (delta 3), Δ2 (delta 2), and Δ1 (delta 1), in order of ascending difficulty. The Δ3 trial types represented trials where one option was a better choice by a difference of three cue values, Δ2 trials represented trials where one option was a better choice by a difference of two cue values, and the Δ1 trial types represented trials where one option was a better choice by a difference of only one cue value. Controlling for difficulty level in each heuristic was also a way to verify that indeed participants were still trying to implement the respective heuristic in each experimental condition. For both the TAL and TTB condition, trials were randomly sampled from the same trial space (see Table 1 and Table 2 in Section B of supplemental material available online for more information on the exact sampling procedure).

The seven cues shown on each trial were economic statistics that would predict whether a developing country would achieve higher Gross Domestic Product (GDP) levels the following year when compared with another developing country (see Section A of supplemental material available online for a list of the statistics). These statistics were artificially created and do not correspond to any real-world data in keeping with our focus on heuristic compliance as opposed to real-world performance. We chose this domain in the hopes that it would be familiar in a general sense while also unlikely to invite prior knowledge to guide decisions (i.e., not strongly interfere with the instructed heuristic use).

#### Procedure

**Figure 1 fig1:**
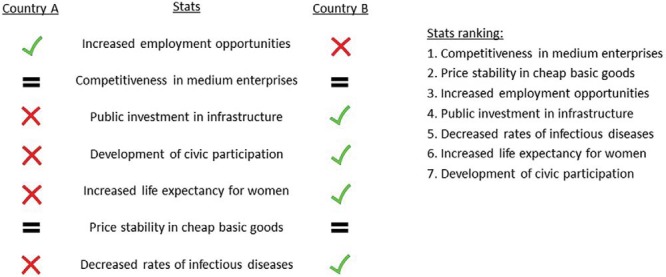
Example trial for the *practice* phase of Experiment 1. Participants were required to choose the country that would have higher gross domestic product (GDP) for the following year depending on the values of the economic statistics presented. Participants were assigned either to the Tallying (TAL) or the Take-the-Best (TTB) condition and asked to respond according to what the respective heuristic would predict. In this example trial, TAL would choose Country B because four cues have superior values (checkmarks) whereas Country A is only superior on one cue. In contrast, TTB would choose Country A because the value for the best-discriminating cue, which in this case is the third most predictive cue (i.e., “Increased employment opportunities”), is superior to Country B.

Subjects were shown the seven cues on each trial and asked to choose which country would have higher GDP the following year. The two options were “Country A” or “Country B.” Participants saw seven economic statistics with a value for each country on each trial (see Figure 1 for an example of stimulus presentation). Each statistic was framed as a comparison between two options (i.e., two developing countries) where a checkmark was presented if superior to the other option, a red cross was presented if inferior to the other option, and a black equal’s sign was presented for ties between countries on a given statistic. A list which ranked the statistics in order of importance, with randomized order between participants, was presented on every trial in the *practice* phase but not in the test phase. Before starting the *practice* phase, participants were provided with detailed instructions on how to use the corresponding heuristic for the condition they had been assigned to (either TAL or TTB).

In the *practice* phase, the participant would see the seven statistics with a value for each country as well as the list that ranked the statistics in order of importance. After making a response, immediate feedback was provided on the screen. If the participant responded in accordance with the heuristic, the screen would show “Good! You understood the rule!”, in addition to an explanation of why the choice was correct according to the heuristic. If the participant did not respond in accordance with the heuristic, the screen would show “Bad. You did not understand the rule.”, in addition to an explanation of why the choice was incorrect according to the heuristic. The statistics with the values for each country were left on screen during feedback but the list of ranked statistics was not. The list was not presented during feedback to discourage reliance on the list and incentivize better engagement with the task. Participants were not directly questioned about their knowledge of the cue validities. The feedback was presented until the participant decided to move on to the next trial followed by a presentation of a blank white screen for 500 ms.

Subsequently, the participant would enter either a block with time pressure or a block without time pressure of the *test* phase. The list which ranked the statistics in order of importance was removed for the *test* phase. For the block without time pressure, the participant was asked to answer in accordance with the heuristic that had been practiced on previously, only this time without feedback. The intertrial interval was 1,500 ms with the first second showing “Thank You!” followed by a presentation of a blank white screen for 500 ms. For the block with time pressure, the participant was also asked to answer in accordance with the heuristic practiced on previously and without feedback. Then, instructions stating that the participant only had 2,000 ms to respond on each trial were provided. The intertrial interval was also 1,500 ms with the first second showing “Thank You!” followed by a presentation of a blank white screen for 500 ms except when the participant reached the 2,000 ms deadline. If the participant reached the 2,000 ms deadline, the screen would explain why it was important for them to adhere to the imposed deadline. This screen was presented for 10,000 ms followed by a presentation of a blank white screen for 500 ms.

### Results

#### Exclusion criteria

Exclusion criteria were based on the responses made on the second half of the *practice* phase (36 trials) and number of missed responses when under time pressure. Participants with performance under 90% in the second half of the *practice* phase or over 16 missed responses when under time pressure were considered outliers and excluded from all subsequent analysis (26 in the TTB condition, 1 in the TAL condition). Including the exclusion criteria from the *practice* phase, this resulted in a total of 26.26% of participants who were excluded from all further analyses in the TTB condition and 1% of participants excluded from the TAL condition. Analyses were also run without any exclusion^1^ and this did not change any conclusions from the analyses shown below. All analyses that follow use people who passed exclusion (*n* = 179) to provide a more stringent evaluation of our predictions. All response time analyses were calculated with median response times. For a presentation of results from the *practice* phase, please refer to Section D in supplemental material available online.

#### Test phase

**Figure 2 fig2:**
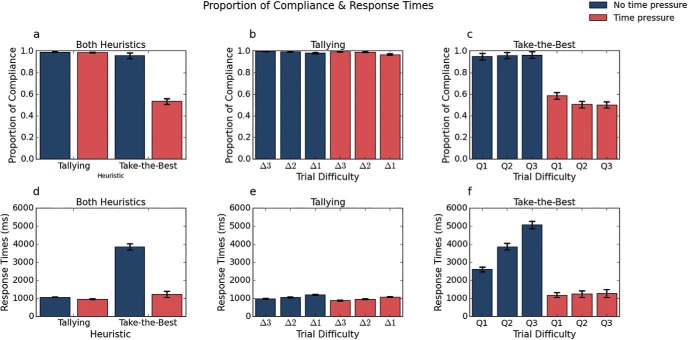
Main results after applying exclusion criteria for Experiment 1 (*n* = 179). The figure shows the proportion of compliance with (a) both heuristics for blocks without time pressure (blue bars) and blocks with time pressure (red bars), (b) the proportion of compliance in the Tallying (TAL) condition for blocks with and without time pressure displayed by degrees of difficulty (deltas), and (c) the proportion of compliance in the Take-the-Best (TTB) condition for blocks with and without time pressure displayed by degrees of difficulty (Qs). Shows the response times for (d) both heuristics in both blocks with and without time pressure, (e) the response times in the TAL condition for blocks with and without time pressure displayed by degrees of difficulty (deltas), and (f) the response times in the TTB condition for blocks with and without time pressure displayed by degrees of difficulty (Qs). For all panels, error bars are 95% within-participants confidence intervals.

TTB participants had lower compliance than TAL, mostly because they were affected more by the time pressure manipulation (see panel A in Figure 2). Proportion of compliance was analyzed using a 2 × 2 × 3 mixed-design analysis of variance (ANOVA) with a between-subjects factor of heuristic (TAL, TTB), a within-participants factor of time pressure (present or absent), and a within-participants factor of trial difficulty (three levels of trial difficulty). Main effects were observed for heuristic, *F*(1, 177) = 285.89, *p* < .001, η^2^ = .62, time pressure, *F*(1, 177) = 352.62, *p* < .001, η^2^ = .67, and trial difficulty, *F*(2, 354) = 13.85, *p* < .001, η^2^ = .07, as well as an interaction between heuristic and time pressure, *F*(1, 177) = 329.37, *p* < .001, η^2^ = .65, an interaction between heuristic and trial difficulty, *F*(2, 354) = 4.53, *p* = .011, η^2^ = .03, an interaction between time pressure and trial difficulty, *F*(2, 354) = 18.66, *p* < .001, η^2^ = .10, and the three-way interaction between heuristic, time pressure, and trial difficulty, *F*(2, 354) = 14.87, *p* < .001, η^2^ = .08. These results directly support our hypothesis by showing that TTB has lower compliance than TAL, especially under time pressure. The three-way interaction highlights the asymmetric effect of the time pressure manipulation on both heuristics’ difficulty levels.[Fig-anchor fig2]

TTB participants responded more slowly than TAL participants and were markedly slower on difficulty trials (see panels D, E, and F in Figure 2). Response times were analyzed using a 2 × 2 × 3 mixed-design ANOVA with a between-subjects factor of heuristic (TAL, TTB), a within-participants factor of time pressure (present or absent) and with a within-participants factor of trial difficulty (three levels of trial difficulty). Individual response times were calculated as median response times. Main effects were observed for heuristic, *F*(1, 177) = 382.41, *p* < .001, η^2^ = .68, time pressure, *F*(1, 177) = 370.92, *p* < .001, η^2^ = .68, and trial difficulty, *F*(2, 354) = 470.60, *p* < .001, η^2^ = .73, as well as an interaction between heuristic and time pressure, *F*(1, 177) = 313.50, *p* < .001, η^2^ = .64, an interaction between heuristic and trial difficulty, *F*(2, 354) = 248.39, *p* < .001, η^2^ = .58, an interaction between time pressure and trial difficulty, *F*(2, 354) = 285.34, *p* < .001, η^2^ = .62, and the three-way interaction between heuristic, time pressure, and trial difficulty, *F*(2, 354) = 265.66, *p* < .001, η^2^ = .60. The main effect of heuristic shows that TTB is a slower heuristic than TAL and directly supports our main hypothesis. The interaction between heuristic and time pressure suggests that TAL can accommodate to time pressure more readily than TTB can. The three-way interaction shows that trial difficulty still shows differences between heuristics in both blocks, with and without time pressure. This gives reassurance that participants were still engaged and attempting to implement the respective heuristic even for blocks with time pressure.

#### Model-based analysis

**Figure 3 fig3:**
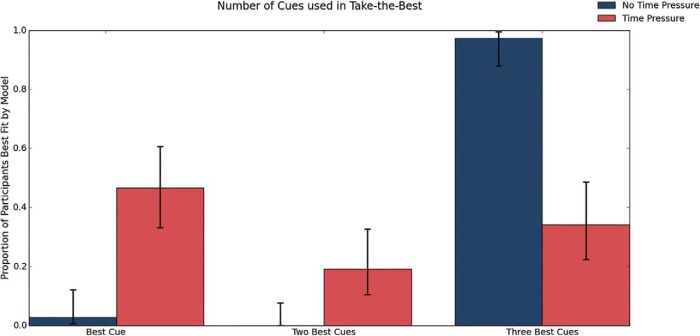
Model-based analyses reveal cue usage for Take-the-Best (TTB) for Experiment 1. The differences in how many cues were used in blocks without time pressure (blue bars) and with time pressure (red bars) for the TTB condition. The vertical axis shows the proportion of participants that were best described by one of the three models. Absent time pressure (blue bars), most participants’ decisions were best fit by the full model using the best three cues, whereas with time pressure (red bars) some participants appeared to rely on fewer cues. Error bars are 95% confidence intervals.

For the TTB participants, three models were fit to determine how many cues participants tended to successfully incorporate under self-paced and speeded conditions. The models are reduced versions of TTB. Only three models were considered; TTB first-cue (only considers the first cue presented on all trials), TTB first-two-cues (only considers the first and second cue presented on all trials), and TTB-first-three-cues (only considers the first, second and third cue presented on all trials). As shown in Figure 3, the number of cues successfully incorporated tended to be higher under self-paced conditions, suggesting that the TTB procedure broke down under time pressure. Fisher’s exact test found that the model contingencies differed across self-paced and speeded conditions (*p* < .001).[Fig-anchor fig3]

Only models with three or fewer cues were considered because only the first three cues were needed to comply with TTB in the study design. Which model best fit each participant was determined by comparing the agreement in choices between human and model. For the one and two cues models, agreement was scored as .5 (i.e., the expectation for a random guess) for trials that exceeded the cues encoded by the model because TTB guesses when there is no discriminating cue. All three models are parameter-free and, thus, are readily comparable. For the speeded blocks, the agreement in choices between human and model were significantly above chance (.5) for the best cue model, *t*(72) = 2.81, *p* = .006, but not for the two best cues model, *t*(72) = 1.68, *p* = .097, or three best cues model, *t(*72) = 1.21, *p* = .229. Additional model fits confirmed that participants were using the cues themselves, as opposed to their positions (see Figure 1 of Section C in supplemental material available online).

### Discussion

Experiment 1 strongly supported our a priori hypothesis: TTB, however frugal, can be slow given its requirements with respect to attentional control and search costs. This can be seen by the lower proportion of compliance of the TTB heuristic under time pressure, whereas the TAL heuristic maintains the same proportion of compliance for both the self-paced and time pressure conditions. We assume that this effect is because of both the trial-by-trial randomization of the cue positions and the format of the values shown (e.g., crosses and checkmarks). These results highlight the importance of considering not only the structure of the environment, but also the cognitive demands of heuristics when pairing heuristics with tasks. Experiment 2 attempts to reverse the TAL advantage observed in Experiment 1 by using a stimulus format that favors TTB by reducing search costs (helpful for TTB) and the possibility of perceptual summation (harmful for TAL).

## Experiment 2

In Experiment 1, TALs advantage over TTB was striking—the less frugal (in terms of cues) heuristic was faster. We predicted this pattern of results by considering the cognitive processes each heuristic requires. In Experiment 2, we attempted to build on this result by creating conditions that should favor TTB over TAL. In Experiment 2, the search costs for TTB are reduced by ordering stimulus cues by their validity. Unlike Experiment 1, the cues are no longer color-coded, which prevents TAL from utilizing perceptual summation operations. Mirroring Experiment 1, the format choice in Experiment 2 should strongly favor TAL over TTB.

### Method

#### Participants

Participants (194 total, 128 female) were also recruited on Amazon Mechanical Turk. Participants were restricted to the United States of America and assigned either to the TAL condition (97 total, 63 female) or the TTB condition (97 total, 65 female). They received $2.50 to complete a 40 min (approx.) learning and decision making task and the best participant was offered a $20 bonus in each condition. The average age of participants was 41.5 years (*SD* = 12.34). The study was approved by the local UCL ethics committee.

#### Design and materials

**Figure 4 fig4:**
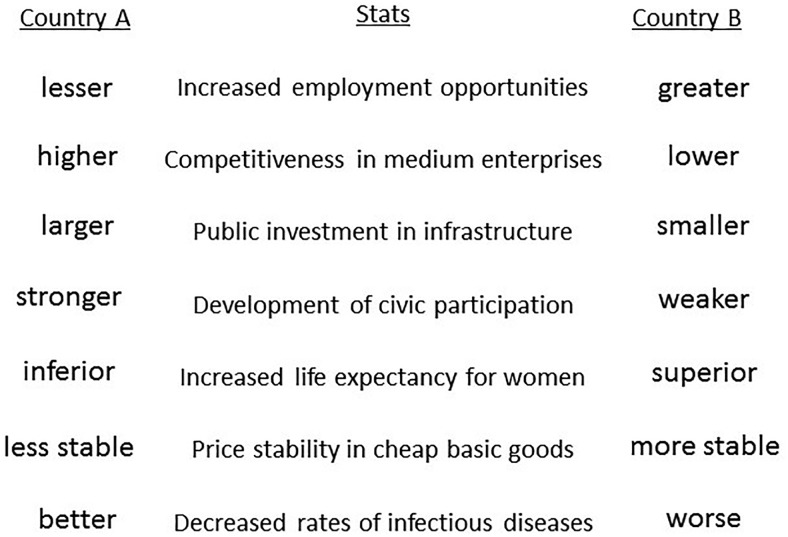
Example trial for the *test* phase of Experiment 2. Participants were required to choose the country that would have higher GDP for the following year depending on the values of the economic statistics presented. Participants were assigned either to the Tallying (TAL) or the Take-the-Best (TTB) condition and asked to respond according to what the respective heuristic would predict. In this example trial, TAL would choose Country A because four cues have superior values (positive adjectives) whereas Country B is only superior on three cues. In contrast, TTB would choose Country B because the value for the best-discriminating cue, which in this case is also the most predictive cue (i.e., “Increased employment opportunities”), is superior to Country A.

The design and materials for the experiment were similar to Experiment 1 with some critical differences. Rather than order cues randomly on each trial, cues were always ordered by their validity. Although the stimuli consisted of the same economic statistics as in Experiment 1, they were no longer color-coded (see Figure 4). Instead, pairs of positive and negative adjectives (e.g., better and worse) were used (see Section A of supplemental material available online for a list of the adjectives). The word “equal” was presented for ties.[Fig-anchor fig4]

#### Procedure

As in Experiment 1, participants were shown the seven cues on each trial and asked to choose which country (“Country A” or “Country B”) would have higher GDP the following year. Participants saw seven economic statistics with a value for each country on each trial (see Figure 4 for an example of stimulus presentation).

### Results

#### Exclusion criteria

Exclusion criteria were the same as in Experiment 1. Participants with performance under 90% in the second half of the practice phase or over 16 missed responses when under time pressure were considered outliers and excluded from all subsequent analysis (8 in the TTB condition, 13 in the TAL condition). Including the exclusion criteria from the practice phase, this resulted in a total of 8.25% of participants who were excluded from all further analyses in the TTB condition and 13.4% of participants excluded from the TAL condition. Analyses were also run without any exclusion^2^ and this did not change any conclusions from the analyses shown below. All analyses that follow use people who passed exclusion (*n* = 173) to provide a more stringent evaluation of our predictions. All response time analyses were calculated with median response times.

#### Test phase

**Figure 5 fig5:**
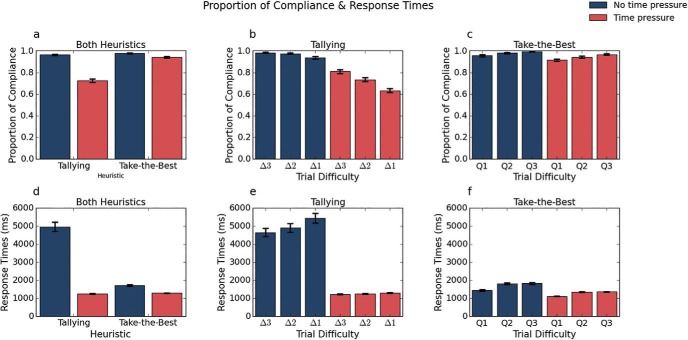
Main results after applying exclusion criteria for Experiment 2 (*n* = 173). The figure shows the proportion of compliance with (a) both heuristics for blocks without time pressure (blue bars) and blocks with time pressure (red bars), (b) the proportion of compliance in the Tallying (TAL) condition for blocks with and without time pressure displayed by degrees of difficulty (deltas), and (c) the proportion of compliance in the Take-the-Best (TTB) condition for blocks with and without time pressure displayed by degrees of difficulty (Qs). Shows the response times for (d) both heuristics in both blocks with and without time pressure, (e) the response times in the TAL condition for blocks with and without time pressure displayed by degrees of difficulty (deltas), and (f) the response times in the TTB condition for blocks with and without time pressure displayed by degrees of difficulty (Qs). For all panels, error bars are 95% within-participants confidence intervals.

TAL participants had lower compliance than TTB, mostly because they were affected more by the time pressure manipulation (see panel A in Figure 5). Proportion of compliance was analyzed using a 2 × 2 × 3 mixed-design ANOVA with a between-subjects factor of heuristic (TAL, TTB), a within-participants factor of time pressure (present or absent), and a within-participants factor of trial difficulty (three levels of trial difficulty). Main effects were observed for heuristic, *F*(1, 171) = 134.83, *p* < .001, η^2^ = .44, time pressure, *F*(1, 171) = 244.74, *p* < .001, η^2^ = .59, and trial difficulty, *F*(2, 342) = 21.51, *p* < .001, η^2^ = .11, as well as an interaction between heuristic and time pressure, *F*(1, 171) = 135.95, *p* < .001, η^2^ = .44, an interaction between heuristic and trial difficulty, *F*(2, 342) = 109.75, *p* = .011, η^2^ = .39, an interaction between time pressure and trial difficulty, *F*(2, 342) = 19.89, *p* < .001, η^2^ = .10, and the three-way interaction between heuristic, time pressure, and trial difficulty, *F*(2, 354) = 29.42, *p* < .001, η^2^ = .15. These results directly support our hypothesis by showing that when search costs are eliminated and the representation of stimuli values obstruct quick summations, then TAL has lower compliance than TTB, especially under time pressure. The three-way interaction highlights the asymmetric effect of the time pressure manipulation on both heuristics’ difficulty levels.[Fig-anchor fig5]

TAL participants responded more slowly than TTB participants and were markedly slower on difficult trials (see panels d, e, and f in Figure 5). Response times were analyzed using a 2 × 2 × 3 mixed-design ANOVA with a between-subjects factor of heuristic (TAL, TTB), a within-participants factor of time pressure (present or absent) and with a within-participants factor of trial difficulty (three levels of trial difficulty). Individual response times were calculated as median response times. Main effects were observed for heuristic, *F*(1, 171) = 171.41, *p* < .001, η^2^ = .50, time pressure, *F*(1, 171) = 291.79, *p* < .001, η^2^ = .63, and trial difficulty, *F*(2, 342) = 110.18, *p* < .001, η^2^ = .39, as well as an interaction between heuristic and time pressure, *F*(1, 171) = 186.14, *p* < .001, η^2^ = .52, an interaction between heuristic and trial difficulty, *F*(2, 342) = 15.80, *p* < .001, η^2^ = .09, an interaction between time pressure and trial difficulty, *F*(2, 342) = 40.50, *p* < .001, η^2^ = .19, and the three-way interaction between heuristic, time pressure, and trial difficulty, *F*(2, 342) = 22.11, *p* < .001, η^2^ = .11. The main effect of heuristic shows that TAL is a slower heuristic than TTB and directly supports our main hypothesis. For this experiment, the interaction between heuristic and time pressure suggests that TTB can accommodate to time pressure more readily than TAL can. The three-way interaction shows that trial difficulty finds differences between heuristics in both blocks, with and without time pressure, which provides reassurance that participants were engaged and attempting to implement the respective heuristic even for blocks with time pressure.

#### Model-based analysis

**Figure 6 fig6:**
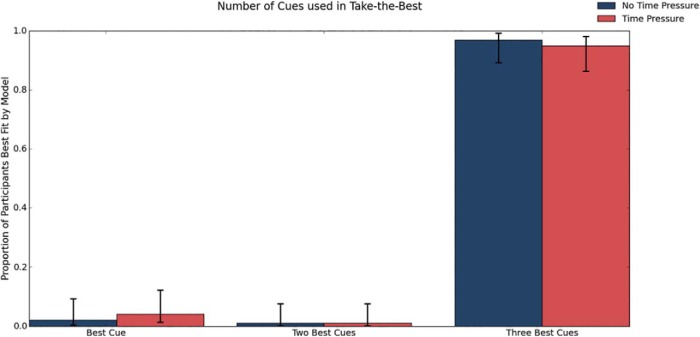
Model-based analyses reveal cue usage for Take-the-Best (TTB). The differences in how many cues were used in blocks without time pressure (blue bars) and with time pressure (red bars) for the TTB condition. The vertical axis shows the proportion of participants that were best described by one of the three models. Most participants’ decisions were best fit by the full model using the best three cues for both speeded and self-paced blocks. Error bars are 95% confidence intervals.

As in Experiment 1, three models were fit for the TTB condition to determine how many cues participants tended to successfully incorporate under self-paced and speeded conditions. As shown in Figure 6, the number of cues successfully incorporated was not different under self-paced conditions, suggesting that the TTB procedure was unaffected by time pressure in this experiment. Fisher’s exact test found that the model contingencies did not differ across self-paced and speeded conditions (*p* = .840). Model agreement with human behavior followed the same rationale as in Experiment 1 (see above).[Fig-anchor fig6]

### Discussion

In accord with our hypothesis, Experiment 2 reversed the effect found in Experiment 1—TAL was more strongly affected by time pressure than TTB. TAL had lower compliance, particularly under time pressure. One interesting side note is that TTB compliance was better for difficult TTB trials, though these trials were also slower which indicates a speed–accuracy trade-off. Overall, the results in Experiment 2 reinforce the conclusions from Experiment 1, namely that heuristics are not monolithic and need to be decomposed into their constituent cognitive processes to understand and predict their performance under various task conditions.

## General Discussion

Heuristics are often contrasted collectively with other decision procedures, which could give the impression that heuristics form a uniform class. Instead, we argued that heuristics are best understood in terms of their constituent cognitive processes. When decomposed into these processes, it becomes possible to predict when people will be successful in applying given the demands of the task.

In Experiment 1, stimulus format was chosen to suite the demands of TAL at the expense of TTB. We found that the more frugal heuristic (i.e., requiring less information), TTB, was slower than the less frugal heuristic, TAL. We hypothesized this advantage was because of the search costs incurred by TTB (cue order was randomized) and the ability of TAL to take advantage of fast perceptual summation operations (cue values were color-coded). In Experiment 2, these advantages and disadvantages were reversed by ordering cues by their validity and removing color-coding. As predicted, we observed an advantage of TTB over TAL.

Our results mirror previous results using different experimental procedures. As in Experiment 1, previous work finds that the time requirements of TTB are dependent on the number of cues retrieved from memory (Bergert & Nosofsky, 2007; Bröder & Gaissmaier, 2007; Khader et al., 2011; Lee & Cummins, 2004; Newell & Shanks, 2003). These results dovetail with the notion that TTB’s implementation involves greater complexity than its algorithmic description suggests (Dougherty et al., 2008; Juslin & Persson, 2002). The findings of Experiment 1 also resemble results of another study where more cue information was processed quicker, as long as the added information would increase coherence among cues (Glöckner & Betsch, 2008). Conversely, participants difficulties with TAL in Experiment 2 mirror previous research exploring when people choose to adopt a compensatory strategy (Bröder, 2003; Bröder & Schiffer, 2006; Platzer & Bröder, 2012; Platzer, Bröder, & Heck, 2014). A surprising result in Experiment 2 was that proportion of compliance for TTB varied with difficulty in the opposite direction of that which was predicted. However, the tradeoff observed between speed and accuracy limits the interpretation of this result (e.g., Ratcliff & McKoon, 2008).

Our results support the view that heuristics should be unpacked in terms of the psychological processes they rely upon. One important area for continued research is understanding how the representational format of cues and their values affect performance (Bröder & Schiffer, 2006; Gigerenzer & Hoffrage, 1995). Ideally, this research would expand to consider other heuristics and to conditions under which participants must choose which strategy to adopt for a given environment (Rieskamp & Otto, 2006). One possibility is that participants are adaptive and choose the heuristic procedure that minimizes the cognitive demands given the stimulus format. The set of candidate solution procedures could extend beyond heuristic procedures to include gist representations (Peters et al., 2009; Reyna, 2008) and parallel constraint satisfaction approaches (Glöckner & Betsch, 2008). Alternatively, there is also recent work suggesting that heuristics can be viewed as a special case of Bayesian inference (Parpart, Jones & Love, under revision).

In contrast to findings suggesting that participants tend to default to strategies similar to TTB under time pressure (Ben Zur & Breznitz, 1981; Payne, Bettman, & Johnson, 1993), we found in Experiment 1 that compliance with TTB was dramatically reduced by time pressure, whereas TAL was largely unaffected. As discussed, our choice of stimuli played an important role in determining these results, as observed when the stimuli were changed in Experiment 2. Additionally, previous research has found that people tend to use strategies more like TTB when the decision is largely made from memory (Bröder & Schiffer, 2003). In contrast, people tend to use strategies more like TAL when the stimulus conveys key information about the options (Bröder & Schiffer, 2003). Our findings, which follow from the predictions of our attentional hypothesis, suggest a possible explanation for previous results. Likewise, our account may help explain the costs of learning TTB.

An important limitation of these two studies is that they differ from past research in that we directly instructed subjects on which strategy to use, either TAL or TTB, and how to use them. For the same reasons as another study (Khader et al., 2011), we preferred to instruct participants to use these heuristics—instead of studying their spontaneous use—as a preventive measure for strategy switching. This detail provides the experimenters with more control over the task structure but it limits both comparison with other studies that do not use instructed strategy use and the interpretability of these results for real-world applications.

In conclusion, more frugal heuristics will not necessarily be faster to implement than less frugal ones. Similarly, less frugal strategies can be fast given the right stimuli format. To understand how heuristics will perform across situations, the cognitive mechanisms that underlie heuristics need to be specified.

In light of these results, one important challenge is matching heuristics to decision environments according to multiple factors, such as stimulus format, time available, and cognitive resources. One practical implication of this line of research is to inform those who aim to best train people, such as emergency responders, to appropriately match decision heuristics to the information and cognitive demands of the task. Although there is much more yet to do, our results make clear that heuristics are not universally “fast and frugal” but in cases may be “fast or frugal.”

## Supplementary Material

10.1037/xlm0000419.supp

## Footnotes

1 We also conducted all analyses with participants that had 100% performance in the second half of the *practice* phase (95 participants in the TAL condition and 25 in the TTB condition) and this did not change any conclusions presented here.

2 We also conducted all analyses with participants that had 100% performance in the second half of the *practice* phase (44 participants in the TAL condition and 48 in the TTB condition) and this did not change any conclusions presented here.
