# Supplementary material for: Fast or Frugal, but Not Both: Decision Heuristics Under Time Pressure
Source: J Exp Psychol Learn Mem Cogn. 2017 May 29;44(1):24–33. doi: 10.1037/xlm0000419 (PMC5708146; doi:10.1037/xlm0000419)
Supplement: Supplementary file 1 [file zfv999173533so1.docx]

**Supplemental Materials**

**Fast or Frugal, but Not Both: Decision Heuristics Under Time Pressure**

**by S. Bobadilla-Suarez & B. C. Love, 2017, *Journal of Experimental Psychology: Learning, Memory, and Cognition***

**http://dx.doi.org/10.1037/xlm0000419**

**Supplemental Material A:** *List of Cues (Statistics for Developing Countries)*

"Competitiveness in medium enterprises",

"Price stability in cheap basic goods",

"Increased employment opportunities",

"Public investment in infrastructure",

"Decreased rates of infectious diseases",

"Increased life expectancy for women",

"Development of civic participation"

**Supplemental Material B:** *Stimuli Sampling Procedure*

Classifying trials based on Take-the-Best difficulty results in Q1, Q2, Q3, Q4, Q5 trials (i.e. trials where looking at the first cue is sufficient, looking at the second is sufficient, etc.). Because only Q1, Q2 & Q3 trials were used, this reduces the space to 468 trials.

Classifying trials based on Tallying difficulty results in trials with Δ1, Δ2, Δ3, Δ4 & Δ5 difference between the amount of negatively-valued (red cross) and positively-valued (green checkmark) cues. Since only Δ1, Δ2 & Δ3 trials were used this reduces the space to 462 trials. Cross-tabulating Tallying & Take-the-Best trial spaces results in a space of 452 trials (Table B1).

| Table B1 | | | | |  |  |
| --- | --- | --- | --- | --- | --- | --- |
| *Cross Tabulation between TAL & TTB Trial Types* | | | | |  |  |
|  |  | **TAL** | | |  |  |
|  |  | *Δ3* | *Δ2* | *Δ1* |  |  |
| **TTB** | *Q1* | 40 | 100 | 180 |  |  |
|  | *Q2* | 10 | 30 | 60 |  |  |
|  | *Q3* | 2 | 8 | 20 |  |  |

In addition to balanced sampling across the two different difficulty classifications, the amount of non-discriminating cues can also affect difficulty in both experimental conditions, (whether participants use TAL or TTB). Trials range from having seven cues with discriminating power down to only three cues with discriminating power. We tried to sample trials with the most diversity possible without repetition of stimuli (Table B2).

| Table B2 | | | | |  |  |
| --- | --- | --- | --- | --- | --- | --- |
| *Cross Tabulation between TAL & TTB Trial Types Sampled* | | | | |  |  |
|  |  | **TAL** | | |  |  |
|  |  | *Δ3* | *Δ2* | *Δ1* | *Total* |  |
| **TTB** | *Q1* | 6 | 3 | 3 | 12 |  |
|  | *Q2* | 5 | 5 | 2 | 12 |  |
|  | *Q3* | 1 | 4 | 7 | 12 |  |
|  | *Total* | 12 | 12 | 12 | 36 |  |

This results in a total of 36 trials that can be partitioned into 3 blocks of Q1, Q2, Q3 trials or equivalently as 3 blocks of Δ3, Δ2, or Δ1 trials respectively. Now adding the trials where the signs are reversed -for right-left presentation instead of left-right presentation of the stimuli- gives a total of 72 trials.

Globally, all 72 trials can also be partitioned into trials where all cues can discriminate between options (n=8), trials where six cues can discriminate between options (n=8), trials where five cues can discriminate between options (n=28), trials where four cues can discriminate between options (n=16) and trials where three cues can discriminate between options (n=12). This can be seen in Table B3.

| Table B3 |  |  |  |  |  |  |  |  |  |  |
| --- | --- | --- | --- | --- | --- | --- | --- | --- | --- | --- |
| *Cross Tabulations of Trial Difficulty with Amount of Non-discriminating Cues for TAL & TTB* | | | | | | | | | | |
| **Tallying** | | | |  |  | **Take-the-Best** | | | |  |
| **# of Equals Signs** | **Δ3** | **Δ2** | **Δ1** | **Total** |  | **# of Equals Signs** | **Q1** | **Q2** | **Q3** | **Total** |
| 0 | 6 | 0 | 2 | 6 |  | 0 | 8 | 0 | 0 | 6 |
| 1 | 0 | 8 | 0 | 8 |  | 1 | 4 | 4 | 0 | 8 |
| 2 | 18 | 0 | 10 | 28 |  | 2 | 8 | 12 | 8 | 28 |
| 3 | 0 | 16 | 0 | 16 |  | 3 | 2 | 6 | 8 | 16 |
| 4 | 0 | 0 | 12 | 12 |  | 4 | 2 | 2 | 8 | 12 |
| **Total** | 24 | 24 | 24 | 72 |  | **Total** | 24 | 24 | 24 | 72 |

**Supplemental Material C:** *Cue Subset Models for TAL & TTB for Experiment 1*

**Subset models for the time pressure phase.** In addition to comparing subjects’ vector of responses to what the respective heuristic predicted for each condition, we also compared these responses to what these heuristics would predict for subsets of the cues. These models were applied exclusively to the time pressure phase as shown in Figure C1. The models were classified in a two by two factorial design (heuristic model x model class), giving four model classes in total for each experimental condition. The first factor consisted of modeling subject responses for TAL subsets (*n* = 128) and for TTB subsets (*n* = 13700) for both conditions to see whether TAL subsets could better account for the data in the TTB condition or vice versa. The second factor (model class) was constructed based on the way cues were defined, either featural or positional. The featural models represent tracking cues based on their names (e.g. "Public investment in infrastructure") and the positional models represent tracking just the positions of the cues (e.g. just looking at the first three cues). A 2x2x2 ANOVA (experimental condition x heuristic model x model class) for five-fold cross-validated predictive accuracy of the models showed a main effect of model class *F*(1,177) = 172.86, *p* < .001, η^2^ = .49, a main effect of heuristic *F*(1,177) = 305.49, *p* < .001, η^2^ = .63, and of their interaction *F*(1,177) = 326.32, *p* < .001, η^2^ = 65. There was also an effect of experimental condition *F*(1,177) = 774.59, *p* < .001, η^2^ = .81, its interaction with heuristic model *F*(1,177) = 233.36, *p* < .001, η^2^ = .57,and the three way interaction of model class with heuristic model and experimental condition *F*(1,177) = 79.15, *p* < .001, η^2^ = .31.


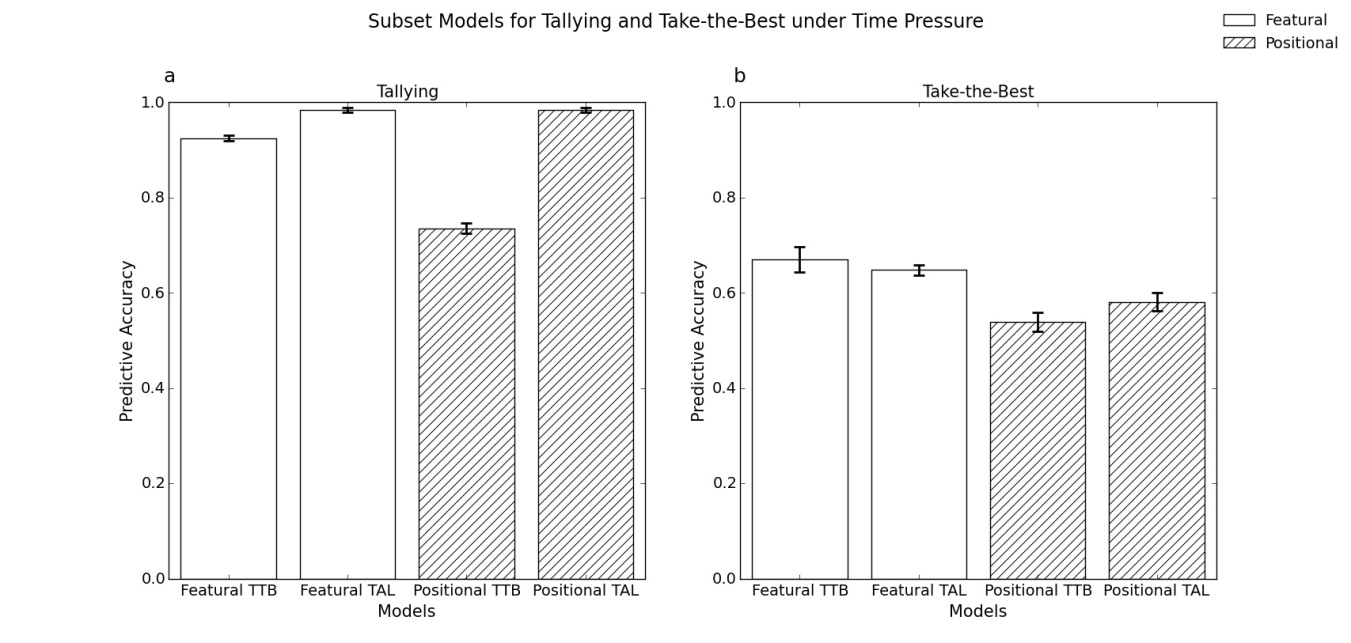


**^Fig. C1.^**

^Subset models under time pressure. On the left is (a) mean predictive accuracy from five-fold cross validation for the four classes of subset models for TAL in the time pressure phase and on the right is (b) the same for TTB. Error bars are within-participant confidence intervals.^

It is important to note that distinguishing between Positional TAL models and Featural TAL models was not possible due to equivalent predictions of both classes of models when subjects were using the full set of cues. We conclude this section with an exposition of the actual models considered for this analysis.

Number of TAL Subset Models:

${\left| TAL \right|=2}^{n}=128$, where $n$ is the total number of cues ($n=7$).

Number of TTB Subset Models: $\left| TTB \right|=\sum_{k=1}^{n=7} \binom{n}{k} k!=13700$

Doubling the number of models to distinguish between positional and featural models, and accounting for duplicates, gives a total of 27639 subset models per subject.

**Supplemental Material D:** *Practice phase analysis for Experiments 1 and 2*


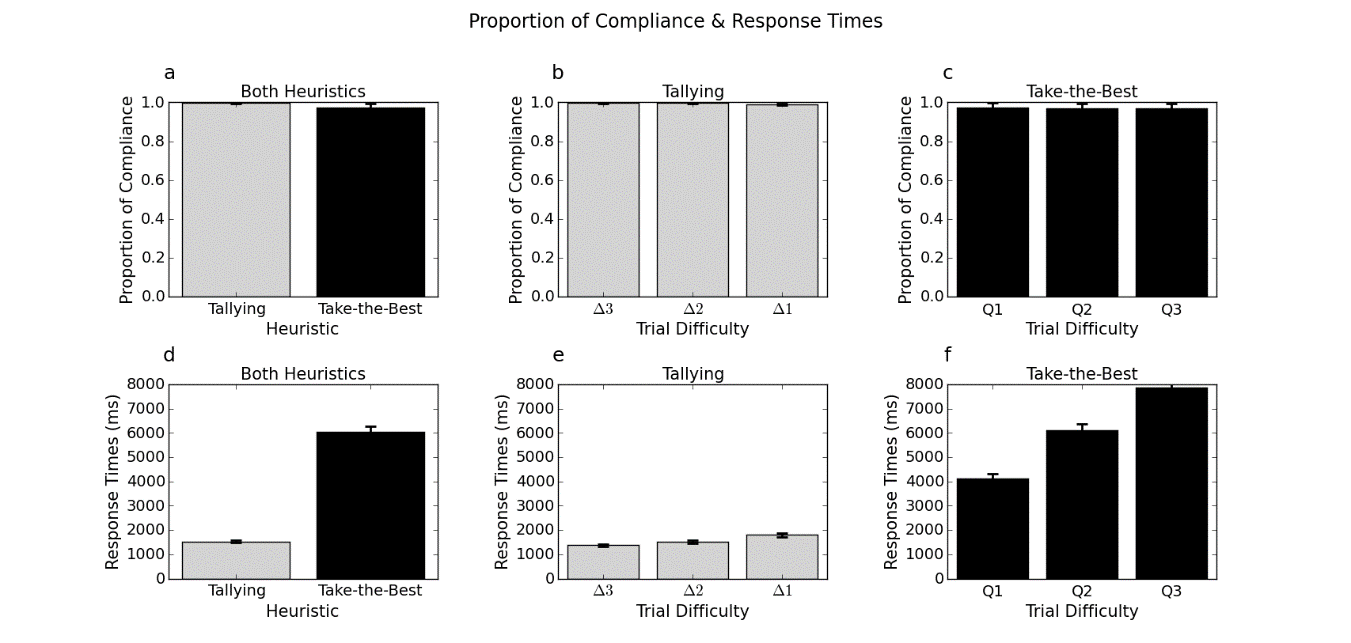


**^Fig. D1.^** ^Results for practice phase of Experiment 1 after applying exclusion criteria (n = 179). The figure shows the proportion of compliance with (a) both heuristics with TAL in gray and TTB in black, (b) the proportion of compliance in the TAL condition displayed by degrees of difficulty (Deltas), and (c) the proportion of compliance in the TTB condition displayed by degrees of difficulty (Qs). Shows the response times for (d) both heuristics, (e) the response times in the TAL condition displayed by degrees of difficulty (Deltas), and (f) the response times in the TTB condition displayed by degrees of difficulty (Qs). For all panels, error bars are 95% within-participants confidence intervals.^

**Experiment 1**. Proportion of compliance was analyzed using a 2x3 mixed-design ANOVA with a between-subject factor of heuristic (TAL, TTB) and a within-participants factor of trial difficulty (3 levels of trial difficulty). Main effects were observed for heuristic, F(1, 177) = 74.29, p < .001, η2 = .296. However, we did not observe effects of trial difficulty F(2, 354) = 1.40, p = .248, η2 = .008, nor of the interaction between trial difficulty and heuristic, F(2, 354) = .767, p = .465, η2 = .004. These results suggest that participants had a slightly harder time learning TTB although both heuristics were close to ceiling (see panels a of Figure D1). Interestingly, trial difficulty was not a relevant factor (with respect to compliance) during the practice phase (see panels b and c of Figure D1).

Response times were analyzed using a 2x3 mixed-design ANOVA with a between-subject factor of heuristic (TAL, TTB) and with a within-participants factor of trial difficulty (3 levels of trial difficulty). Main effects were observed for heuristic, F(1, 177) = 456.69, p < .001, η2 = .721 and trial difficulty, F(2, 354) = 398.59, p < .001, η2 = .692, as well as for the interaction of heuristic and trial difficulty, F(2, 354) = 253.75, p < .001, η2 = .589. These results confirm the fact that TTB was harder to implement in this experiment given the slower response times (see panel d of Figure D1). Furthermore, trial difficulty did not affect response times for TAL (panel e of Figure D1) but did affect response times for TTB (panel f of figure D1).


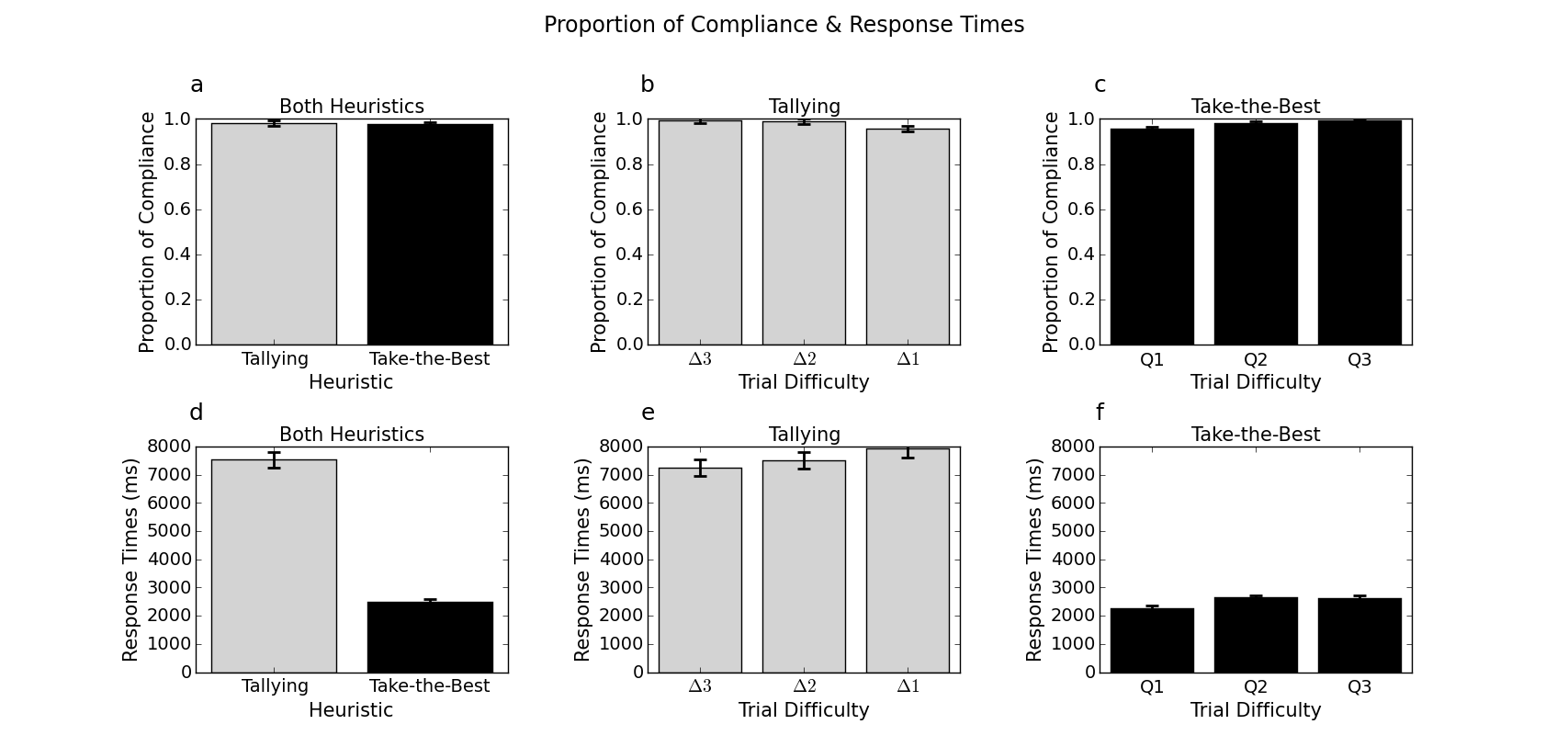


**^Fig. D2.^** ^Results for practice phase of Experiment 2 after applying exclusion criteria (n = 173). The figure shows the proportion of compliance with (a) both heuristics with TAL in gray and TTB in black, (b) the proportion of compliance in the TAL condition displayed by degrees of difficulty (Deltas), and (c) the proportion of compliance in the TTB condition displayed by degrees of difficulty (Qs). Shows the response times for (d) both heuristics, (e) the response times in the TAL condition displayed by degrees of difficulty (Deltas), and (f) the response times in the TTB condition displayed by degrees of difficulty (Qs). For all panels, error bars are 95% within-participants confidence intervals.^

**Experiment 2**. Proportion of compliance was analyzed using a 2x3 mixed-design ANOVA with a between-subject factor of heuristic (TAL, TTB) and a within-participants factor of trial difficulty (3 levels of trial difficulty). Main effects were not observed for heuristic, F(1, 171) = .295, p = .588, η2 = .002. However, we did observe effects of trial difficulty F(2, 342) = 5.87, p = .003, η2 = .033, and of the interaction between trial difficulty and heuristic, F(2, 342) = 60.59, p < .001, η2 = .26. These results resemble the fact that participants adequately learned the use of both heuristics and that trial difficulty affected their learning (see panels a, b and c of Figure D2). The fact that TTB shows better compliance with harder trials is unexpected but is also in line with the results seen during the test phase.

Response times were analyzed using a 2x3 mixed-design ANOVA with a between-subject factor of heuristic (TAL, TTB) and with a within-participants factor of trial difficulty (3 levels of trial difficulty). Main effects were observed for heuristic, F(1, 171) = 309.58, p < .001, η2 = .644 and trial difficulty, F(2, 342) = 21.33, p < .001, η2 = .111, as well as for the interaction of heuristic and trial difficulty, F(2, 342) = 3.94, p = .02, η2 = .022. These results confirm the fact that TAL was harder to implement in this experiment given the slower response times (see panel d of Figure D2).
